# Acute respiratory distress syndrome relapsing in 10 months with an initial manifestation of polymyositis

**DOI:** 10.1002/ccr3.5147

**Published:** 2021-12-06

**Authors:** Yukihisa Takeda, Mariko Ono, Hayato Kinoshita, Yoko Nagatomo, Hiroki Miyauchi, Hiroshi Tsukamoto, Hiroyuki Nakamura, Kazutetsu Aoshiba

**Affiliations:** ^1^ Department of Respiratory Medicine Tokyo Medical University Ibaraki Medical Center Ibaraki Japan; ^2^ Department of Respiratory Medicine Tokyo Medical University Tokyo Japan; ^3^ Department of Neurology Tokyo Medical University Ibaraki Medical Center Ibaraki Japan

**Keywords:** acute respiratory distress syndrome, coronavirus disease 2019, interstitial lung disease, polymyositis

## Abstract

Autoimmune disorders are an important cause of acute respiratory distress syndrome (ARDS). We report a case of a patient with steroid‐responsive ARDS that relapsed in 10 months with an initial manifestation of seronegative polymyositis. ARDS associated with polymyositis may develop earlier than myopathy and may relapse later.

## Graphical abstract

The differential diagnosis of acute respiratory distress syndrome without clear etiology should include a pulmonary manifestation of polymyositis and dermatomyositis, which may develop earlier than myopathy and may relapse later.

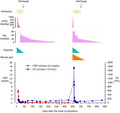

## 1 INTRODUCTION

Physicians in the coronavirus disease 2019 (COVID‐19) pandemic can encounter patients with acute respiratory distress syndrome (ARDS) with challenging differential diagnosis more frequently. A recent study showed that 8% of patients with ARDS had no common risk factors, such as pneumonia, sepsis, aspiration of gastric contents, major trauma, and pancreatitis.^1^ One of the important causes of such atypical ARDS is interstitial lung disease (ILD) associated with an autoimmune disorder. ILD is an important extra‐muscular manifestation of idiopathic inflammatory myopathies (IIM), such as PM and dermatomyositis (DM).^2^, ^3^, ^4^ It occurs in 41% of the patients with PM and DM, with a higher prevalence of ILD in Asia (50%) than in America (23%) and Europe (26%).^5^ The severity of ILD in PM/DM varies from asymptomatic chronic pulmonary fibrosis to an acute and rapidly progressive respiratory failure.^2^ Among them, the acute and rapidly progressive form of ILD, which often represents a life‐threatening condition with ARDS, is frequently seen in patients that are positive for anti‐MDA5 antibodies and some types of anti‐ARS antibodies, such as PL‐7 and PL‐12.^2^, ^6^, ^7^, ^8^ We report a case of a patient presenting with steroid‐responsive ARDS that relapsed in10 months with an initial manifestation of polymyositis (PM).

## 2 CASE PRESENTATION

**FIGURE 1:**
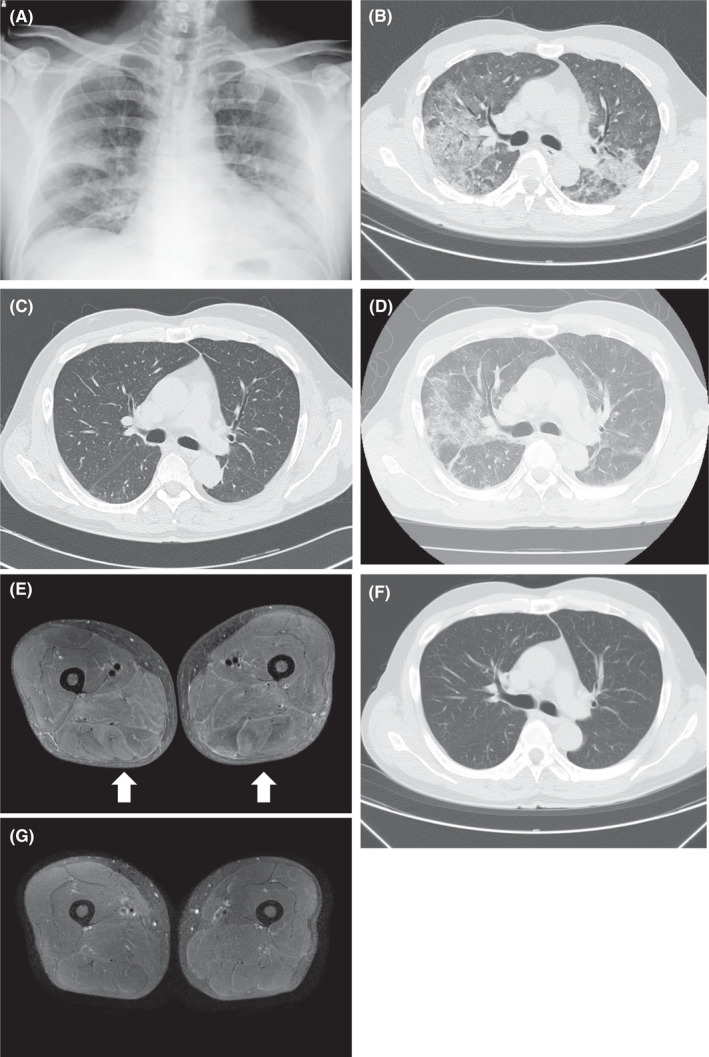
Radiologic findings of the patient. Chest X‐ray (A) and plain chest computed tomography (CT) (B) images taken on the first day of admission showed diffuse ground‐glass opacification and consolidation in bilateral lung fields. (C) A plain chest CT image taken on the 13th day of the first admission showed the disappearance of abnormal shadows with minimal scarring. (D) A plain chest CT image taken on the first day of the 2nd admission demonstrated a relapse of diffuse ground‐glass opacification and consolidation in bilateral lung fields. (E) The short‐tau inversion recovery (STIR) of magnetic resonance imaging (MRI) on the 3rd day of the 2nd admission showed a high signal in the hamstring muscles (*arrows*), which reflects muscle edema and inflammation. (F) A plain chest CT image on the 17th day of the 2nd admission showed resolution of abnormal shadows. (G) The STIR of MRI taken on Day 24 of the second admission showed resolution of high signal in the hamstring muscles

**FIGURE 2:**
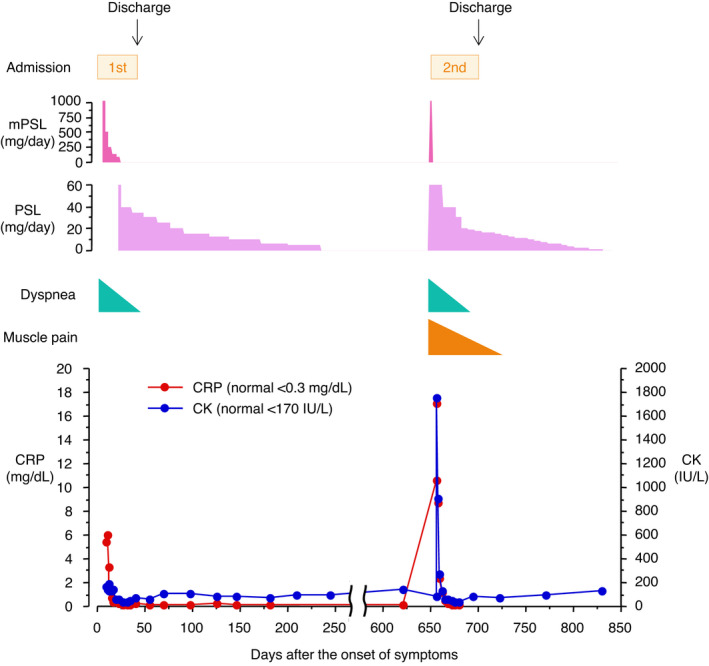
Timeline of the patient's clinical course. Abbreviations: mPSL, methylprednisolone; PSL, prednisolone; CRP, C‐reactive protein; CK, creatinine kinase

A 60‐year‐old Japanese male patient without any past medical history presented with dyspnea for 5 days in June 2019 (before the COVID‐19 outbreak). He had no history of cigarette smoking, alcohol consumption, or sick contacts. He had a frequent cough, tachypnea (40 breaths per minute), low‐grade fever (37.2°C), and hypoxemia (PaO_2_, 50.2 mm Hg on room air). He did not have wheezes or lung crackles and abnormal heart sounds on auscultation. Edema, skin rash, muscle weakness, myalgia, and arthralgia were absent. Blood tests revealed leukocytosis (10,300 cells/μl with 76% neutrophils, 2.0% eosinophils, and 14.0% lymphocytes) with high C‐reactive protein levels (5.27 mg/dl). He had normal liver and renal function tests (aspartate aminotransferase 25 IU/L, normal <38 IU/L; alanine aminotransferase 30 IU/L, normal <40 IU/L; blood urea nitrogen 13.6 mg/dl, normal <20 mg/dl; and creatinine 1.04 mg/dl, normal <1.10 mg/dl) and no elevation of creatinine kinase (155 IU/L, normal <170 IU/L). Autoimmune screening did not identify any abnormalities, including anticyclic citrullinated peptide, anti‐nuclear antibodies, anti‐double‐stranded DNA antibodies, anti‐proteinase 3 (PR3) antibodies, anti‐myeloperoxidase (MPO) antibodies, anti‐Scl‐70 antibodies, anti‐Sjögren's syndrome‐related antigen A (SSA/Ro52) antibodies, anti‐aminoacyl‐transfer RNA synthetase (ARS) antibodies, anti‐Jo‐1 antibodies, and anti‐melanoma differentiation‐associated gene 5 (MDA5) antibodies. Chest X‐ray and computed tomography (CT) scan showed diffuse ground‐glass opacification and consolidation in bilateral lung fields (Figure 1A and B). On the day of admission, the patient's condition deteriorated rapidly and he received noninvasive intermittent positive pressure ventilation (NPPV). The diagnosis of rapidly progressive interstitial lung disease with autoimmune disorders, such as severe inflammatory myopathy‐related interstitial lung disease, could not be ruled out. Based on the diagnosis of ARDS of unknown etiology (PaO_2_/FiO_2_ 235 with a positive end‐expiratory pressure of 5 cmH_2_O), high‐dose (1000 mg/day) intravenous (IV) methylprednisolone therapy was initiated. Empiric antibiotics (IV piperacillin‐tazobactam and levofloxacin) were also given, although blood culture and urinary pneumococcal and Legionella antigen tests were negative. After 3 days of steroid pulse therapy, the patient improved dramatically and was weaned from NPPV and, thereafter, from oxygen support. The dose of IV methylprednisolone was reduced to half every 3 days and later it was switched to oral prednisolone (60 mg/day), which was also gradually reduced. Chest CT scan taken on the 13th day of admission revealed almost complete disappearance of abnormal shadows from the lung field (Figure 1C). He was discharged without dyspnea on the 26th day of admission. The steroid was tapered down gradually and discontinued 7 months after discharge. Although the patient had been asymptomatic for a while, he had a relapse of dyspnea in 10 months later after the first onset of ARDS. Upon the second admission, he had hypoxemia (PaO_2_ 64 mm Hg) on O_2_ 5L/min via face mask and started receiving NPPV therapy. Chest CT scan showed a mixture of diffuse ground‐glass opacification and consolidation similar to roentgenological patterns observed previously (Figure 1D). The findings of physical examination and blood tests were not significantly different from the previous admission except that he had grasping pain in both thighs, proximal muscle weakness in extremities, and elevation of serum creatinine kinase (1741 IU/L). He had no skin eruptions, such as nail‐bed telangiectasia, heliotrope rash, Gottron's papules, Raynaud's phenomenon, and hyperkeratotic lesions on his fingers (mechanic's hands). The short‐tau inversion recovery sequence (STIR) of magnetic resonance imaging (MRI) showed inflammatory changes in both hamstring muscles (Figure 1E). However, the Euroline myositis line blot assay showed negative results for either myositis‐specific antibodies (Jo‐1, PL‐7, PL‐12, EJ, SRP, Mi‐2, MDA5, and TIF1‐γ) or myositis‐associated antibodies (Ku, PM‐Scl100, Scl‐70, and SSA/Ro52). From these findings, the diagnosis of ARDS that relapsed along with an initial manifestation of seronegative PM was made. After 3 days of high‐dose (1,000 mg daily) IV methylprednisolone therapy, the patient's dyspnea and muscle weakness improved dramatically and NPPV therapy was discontinued. Additionally, the diffuse abnormal shadows on the chest CT scan (Figure 1F) and the high signal on STIR MRI of the hamstring muscles (Figure 1G) disappeared. The dose of IV methylprednisolone was gradually reduced to 40 mg/day prednisolone, when the patient was discharged without respiratory and muscular symptoms on the 26th day of the second admission (Figure 2). The steroid was tapered down gradually and discontinued 6 months after discharge.

## 3 DISCUSSION

This patient had several unique clinical features. First, he showed a good clinical response to steroid pulse therapy. It is reported that patients with PM/DM developing diffuse alveolar damage, which largely corresponds to the histopathology of ARDS, have a poor prognosis and sometimes fatal consequences despite high‐dose steroid therapy.^3^, ^9^ Since the steroid therapy led to the complete resolution of both respiratory and muscular symptoms in this patient, we suspect that ARDS resulted from a systemic inflammatory response, which involved the lungs and muscles, and not from diffuse alveolar damage to the lungs. Second, ARDS preceded the manifestation of myopathy, which became evident when ARDS relapsed 10 months later. ILD in PM/DM can precede or follow myopathy or occur simultaneously with it.^10^ However, recurrent and remittent ARDS preceding the diagnosis of DM/PM is rare because of a potentially poor prognosis of ARDS. In this context, patients with anti‐ARS antibodies have been shown to respond well to steroid therapy, but with frequent disease recurrence.^3^, ^11^ Third, this patient had no identifiable autoantibodies on the Euroline myositis line blot assay; however, the result does not exclude the diagnosis of PM because of the low prevalence of autoantibodies in IIM, as exemplified by a recent study showing that 38% of the patients with IIM were autoantibody‐negative.^12^ Further studies might identify new autoantigenic targets in patients with PM/DM who are currently regarded as autoantibody‐negative.^6^

The differential diagnosis of ARDS should include a pulmonary manifestation of PM/DM, which may precede myopathy symptoms. ARDS without clear etiology could be a manifestation of a latent form of PM/DM despite the seronegative profile.

## CONFLICT OF INTEREST

The authors have no conflicts of interest to declare.

## AUTHOR CONTRIBUTIONS

YT and KA wrote the first draft. MO, HK, YN, HM, HT, and HN revised the manuscript.

## CONSENT

Written consent from the patient was obtained for submission and publication of the case details and images.

## Data Availability

The data that support the findings of this study are available on request from the corresponding author. The data are not publicly available due to privacy or ethical restrictions.
